# Characteristics of Spondylotic Myelopathy on 3D Driven-Equilibrium Fast Spin Echo and 2D Fast Spin Echo Magnetic Resonance Imaging: A Retrospective Cross-Sectional Study

**DOI:** 10.1371/journal.pone.0100964

**Published:** 2014-07-15

**Authors:** Mike A. Abdulhadi, Joseph R. Perno, Elias R. Melhem, Paolo G. P. Nucifora

**Affiliations:** 1 Children's Hospitals and Clinics of Minnesota, Minneapolis, Minnesota, United States of America; 2 Philadelphia VA Medical Center, Philadelphia, Pennsylvania, United States of America; 3 University of Maryland, Baltimore, Maryland, United States of America; 4 University of Pennsylvania, Philadelphia, Pennsylvania, United States of America; Glasgow University, United Kingdom

## Abstract

In patients with spinal stenosis, magnetic resonance imaging of the cervical spine can be improved by using 3D driven-equilibrium fast spin echo sequences to provide a high-resolution assessment of osseous and ligamentous structures. However, it is not yet clear whether 3D driven-equilibrium fast spin echo sequences adequately evaluate the spinal cord itself. As a result, they are generally supplemented by additional 2D fast spin echo sequences, adding time to the examination and potential discomfort to the patient. Here we investigate the hypothesis that in patients with spinal stenosis and spondylotic myelopathy, 3D driven-equilibrium fast spin echo sequences can characterize cord lesions equally well as 2D fast spin echo sequences. We performed a retrospective analysis of 30 adult patients with spondylotic myelopathy who had been examined with both 3D driven-equilibrium fast spin echo sequences and 2D fast spin echo sequences at the same scanning session. The two sequences were inspected separately for each patient, and visible cord lesions were manually traced. We found no significant differences between 3D driven-equilibrium fast spin echo and 2D fast spin echo sequences in the mean number, mean area, or mean transverse dimensions of spondylotic cord lesions. Nevertheless, the mean contrast-to-noise ratio of cord lesions was decreased on 3D driven-equilibrium fast spin echo sequences compared to 2D fast spin echo sequences. These findings suggest that 3D driven-equilibrium fast spin echo sequences do not need supplemental 2D fast spin echo sequences for the diagnosis of spondylotic myelopathy, but they may be less well suited for quantitative signal measurements in the spinal cord.

## Introduction

Magnetic resonance imaging (MRI) is commonly used to assess chronic neck pain because of its relative safety as well as the excellent contrast it provides for osseous structures, neural tissue, and cerebrospinal fluid [1]. A typical MRI examination is composed of multiple sequences, each of which is directed towards different clinical questions. In the evaluation of cervical spondylosis, an MRI examination often includes multiple T2-weighted sequences that can assess the degree of degenerative spinal stenosis as well as detect the presence of cord pathology such as spondylotic myelopathy [2], [3]. These sequences generally use T2*-weighted gradient-recalled echo techniques or T2-weighted fast spin echo techniques.

Gradient-recalled echo techniques typically have a short repetition time (TR) due to their lack of a refocusing pulse. This results in faster acquisition time that can be leveraged to perform 3D imaging and improve resolution [4]–[7]. However, gradient-recalled echo techniques are prone to artifact from magnetic susceptibility that may cause image degradation near osseous structures such as the spine, aliasing in the slice select direction, and limited signal-to-noise ratios in comparison to spin echo sequences [4]–[10].

Fast spin echo techniques tend to have longer TR than gradient-recalled echo sequences, even with the use of echo trains to reduce overall scan time. Consequently, conventional fast spin echo techniques are more prone to motion artifact and generally unsuitable for 3D imaging. Driven-equilibrium pulses, when applied to fast spin echo sequences, use a resonant 90-degree radiofrequency pulse to reduce the time needed to transform residual transverse magnetization into longitudinal magnetization [11]. The addition of this pulse to a fast spin echo sequence can shorten TR sufficiently to make 3D acquisition feasible at high resolution [12]–[14].

Apart from reduced scan times, driven-equilibrium fast spin echo sequences have radiographic features that make them well-suited for imaging the cervical spine. A complex combination of T2 and T1 tissue signal characteristics results in a "myelographic effect" that may facilitate evaluation of the cervical spinal canal [5], [6], [12]–[14]. Near-isotropic voxel dimensions allow radiologists to perform multiplanar reformation, resulting in improved visualization of the vertebral bodies, intervertebral disks, posterior longitudinal ligaments, facet joints, and uncovertebral joints [5], [6], [15].

Many lesions in the cervical spinal cord are inconspicuous, small, and prone to partial volume effects [16]. Thus, 3D driven-equilibrium fast spin echo (3D-FSE) sequences, which typically use relatively thin slices, could potentially detect cord lesions as well as conventional 2D fast spin echo (2D-FSE) sequences. However, to our knowledge the appearance of cord lesions on these two sequences has not been directly compared. If they were equivalent, then 3D-FSE sequences could replace 2D-FSE sequences, total scan time could be reduced, and patient comfort could improve. In this work, we address the hypothesis that the appearance of spondylotic cord lesions in the cervical spine is equivalent on 2D-FSE and 3D-FSE sequences.

## Materials and Methods

### Ethics statement

All study protocols were approved by the institutional review board of the Philadelphia VA Medical Center. Waiver of informed consent was obtained due to the retrospective nature of this study. Patient records were de-identified prior to analysis.

A retrospective imaging review was performed for 30 adult patients consecutively diagnosed with spondylotic myelopathy over a six month period. In each of these cases, patients had undergone a clinical MRI examination of the cervical spine using both 2D-FSE and 3D-FSE sequences. These patients had no history of demyelination, spinal neoplasm, spinal infarction, or spinal infection.

All imaging was performed on a single 1.5 tesla scanner (Siemens Avanto). 2D-FSE images were acquired in the axial plane (Repetition time  = 4310 ms, echo time  = 99 ms, slice thickness  = 3 mm, field of view  = 200 mm, matrix dimensions  = 200×256, echo train length  = 15, acquisition time  = 4∶26) using GRAPPA parallel imaging with a reduction factor (R) of 2. 3D-FSE images were acquired in the sagittal plane and reformatted in the axial plane (Repetition time  = 1200 ms, echo time  = 119 ms, slice thickness  = 0.9 mm, field of view  = 280 mm, matrix dimensions  = 320×320, acceleration factor  = 3, echo train length  = 69, acquisition time  = 5∶53, voxel dimensions  = 0.9 mm×0.9 mm×0.9 mm) using GRAPPA parallel imaging with a reduction factor (R) of 3.

Regions of interest (ROIs) were manually placed on cord lesions by a rater without knowledge of the medical record. Evaluation sessions for 2D-FSE and 3D-FSE images were scheduled separately in order to reduce recall bias. For each lesion, in-plane axial dimensions, area, and location were recorded. Within the lesion ROIs, mean signal intensity (S_lesion_) was measured. Within one level of the lesion, mean signal intensity of normal cord (S_normal cord_) was measured. Finally, mean signal in a region of air was measured in each study. These measurements were used to calculate the contrast-to-noise ratio (CNR) for each lesion, defined as:
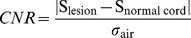
where σ_air_ is the standard deviation of air signal intensity measurements [5], [6].

Lesions were cross-referenced by location and classified into three groups depending on the sequences that depicted them. Lesions visible both on 2D-FSE images and on 3D-FSE images were classified as “type 1”. Lesions visible only on 2D-FSE images were classified as “type 2”. Lesions visible only on 3D-FSE images were classified as “type 3”.

Comparisons of mean lesion dimensions, mean lesion area, and CNR were performed using the unpaired Student's t-test. Cohen's d was calculated for comparisons meeting the significance threshold (two-tailed p<0.05 with Bonferroni correction for multiple comparisons).

## Results

**Figure 1:**
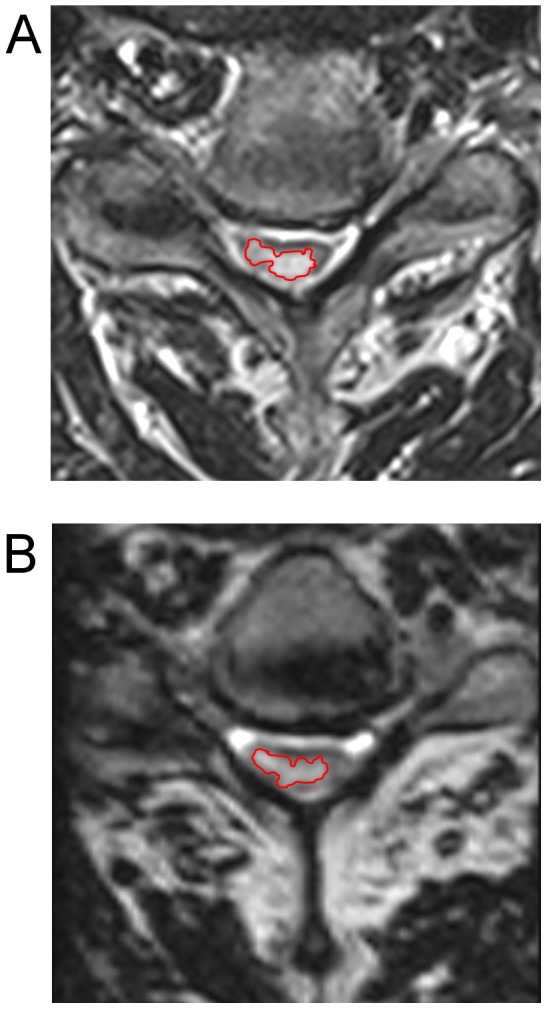
Example of cord lesion in the cervical spine. Images of the cervical spine in a representative patient demonstrated a region of abnormal cord signal both on (a) 2D-FSE sequences and (b) 3D-FSE sequences. The lesion was qualitatively similar on both sequences and approximately the same size. However, it appeared slightly less hyperintense on 3D-FSE images than on 2D-FSE images. A red outline illustrates the typical placement of the ROIs used for analysis.

The study population consisted of 27 men and 3 women with a mean age of 61 years. Qualitatively, the appearance of cord lesions was fairly similar on 2D-FSE and 3D-FSE sequences (see Figure 1).

We found no significant difference between 2D-FSE and 3D-FSE sequences in the mean number of lesions detected (2.0 lesions per patient and 2.2 lesions per patient respectively, uncorrected p>0.05). The majority of these were type 1 lesions (1.4 lesions per patient). There was no significant difference in the mean number of type 2 lesions on 2D-FSE sequences and the mean number of type 3 lesions on 3D-FSE sequences (0.6 lesions per patient and 0.8 lesions per patient respectively, uncorrected p>0.05).

There was no significant difference between 2D-FSE and 3D-FSE sequences in mean lesion dimensions or mean lesion area (see Table 1). However, mean CNR was greater on 2D-FSE sequences than on 3D-FSE sequences (d = 1.6, corrected p<0.001). This was a robust finding that persisted when considering only type 1 lesions (d = 1.5, corrected p<0.001) or when comparing type 2 to type 3 lesions (d = 1.8, corrected p<0.01).

**Table 1:** Summary of cord lesion measurements.

		Type 1		Type 2		Type 3		All types	
		mean	sd	mean	sd	mean	sd	mean	sd
2D-FSE	long axis (mm)	4.2	2.3	3.6	1.5	N/A	N/A	4.0	2.1
	short axis (mm)	2.4	0.9	2.1	0.9	N/A	N/A	2.3	0.9
	area (mm^2^)	8.5	6.3	5.9	3.8	N/A	N/A	7.7	5.8
	CNR	44.1*	17.1	50.4**	24.8	N/A	N/A	45.9***	19.6
3D-FSE	long axis (mm)	4.6	2.4	N/A	N/A	4.1	2.9	4.4	2.6
	short axis (mm)	2.6	1.2	N/A	N/A	2.0	0.8	2.4	1.1
	area (mm^2^)	9.5	8.9	N/A	N/A	6.7	5.9	8.5	8.0
	CNR	22.5*	10.9	N/A	N/A	20.1**	6.8	21.6***	9.6

Mean and standard deviation (sd) for measurements on 2D-FSE and 3D-FSE sequences of Type 1 lesions, Type 2 lesions (2D-FSE only), Type 3 lesions (3D-FSE only), and all lesions.

(*) corrected p<0.001.

(**) corrected p<0.01.

(***) corrected p<0.001.

On 2D-FSE sequences, there was no significant difference between type 1 and type 2 lesions in mean lesion dimensions, mean lesion area, or mean CNR. On 3D-FSE sequences, there was no significant difference between type 1 and type 3 lesions in mean lesion dimensions, mean lesion area, or mean CNR.

## Discussion

We found no significant differences in the number of lesions detected using 2D-FSE and 3D-FSE sequences. Likewise, the dimensions and area of cord lesions did not differ significantly. Since these are the characteristics that are most often used to characterize cord lesions in a clinical setting, this study offers support for the equivalence of these sequences when evaluating cord signal in spondylotic myelopathy. Nevertheless, a caveat is presented by our measurements of CNR, which significantly favored 2D-FSE sequences.

A possible explanation for the differences in CNR may lie in the complex character of the signal in 3D-FSE sequences, which depends on both T1 and T2 [5], [6], [14]. This produces strong contrast between cerebrospinal fluid and the spinal cord in 3D-FSE sequences, but may also contribute to reduced contrast in spinal cord lesions. While differences in CNR had no observable effects on lesion detection in our study, this may be a more important factor in situations that require quantitative signal analysis.

Cord signal change is rare in asymptomatic patients but more common in patients presenting for preoperative evaluation of cervical stenosis [17]–[19]. Abnormal cord signal on MRI is strongly correlated to cord injury on histopathologic evaluation [16], [20]. Furthermore, a recent study suggested that intraparenchymal T2 hyperintensity in spondylotic cord lesions is correlated to the presence of abnormalities on physical examination [21]. This has been corroborated by preoperative comparisons of the clinical condition of patients with and without cord signal abnormality [20]. However, other studies have not shown a relationship between clinical severity and findings on cervical spine MRI [22], [23]. MRI of the cervical spine may play a more important role as a prognostic tool. Many studies have suggested that the appearance of cord lesions on MRI can predict clinical improvement after cervical spine decompression [20], [24]–[36], particularly if cord signal improves after surgery, although a few studies have raised some doubts [23], [37]–[39].

Our study is limited by several factors. First, we calculated CNR based on noise measured in air, outside of the lesion ROI. However, the parallel imaging techniques we used may cause inherent spatial variation in the distribution of noise, resulting in uncertainty in the accuracy of lesion CNR [4]. Although the sequence parameters we used are typical of those used in practice, it is possible that different results could have been obtained with different sequence parameters or sampling regions.

Because of the retrospective nature of this study, we were not able to assess clinical outcomes in the patients we examined. Thus, we cannot determine which findings are best correlated to current or future neurological deficits. Due to practical considerations, we did not obtain a histopathologic reference standard for cord lesions, and thus we cannot determine the diagnostic accuracy of our imaging findings. Instead, we classified lesions according to their visibility on 2D-FSE and 3D-FSE images. Type 1 lesions, visible on both sequences, are most likely to represent “true positives”. Type 2 and type 3 lesions, each visible on only one sequence, could represent either “false positives” on the sequence that depicted them or “false negatives” on the sequence that did not depict them. Regardless, type 2 and type 3 lesions only accounted for about a third of all detected cord lesions, confirming that cord signal change is generally a reproducible finding [40].

Finally, as this was a study of patients with radiologically identified cord lesions, we could not determine whether the differences we observed in CNR would affect miss rates in clinical practice. These important issues should be further evaluated in future prospective studies. We expect that continued development of dedicated sequences for the cervical spine will improve both the diagnostic and prognostic value of MRI in spondylotic myelopathy.

## References

[1] Tracy JA, Bartleson JD (2010) Cervical spondylotic myelopathy. Neurologist 16: 176–187. 20445427 10.1097/NRL.0b013e3181da3a29

[2] Maus TP (2012) Imaging of spinal stenosis: neurogenic intermittent claudication and cervical spondylotic myelopathy. Radiol Clin North Am 50: 651–679. 22643390 10.1016/j.rcl.2012.04.007

[3] Ramanauskas WL, Wilner HI, Metes JJ, Lazo A, Kelly JK (1989) MR imaging of compressive myelomalacia. J Comput Assist Tomogr 13: 399–404. 2723169 10.1097/00004728-198905000-00005

[4] Meindl T, Wirth S, Weckbach S, Dietrich O, Reiser M, et al (2009) Magnetic resonance imaging of the cervical spine: comparison of 2D T2-weighted turbo spin echo, 2D T2*weighted gradient-recalled echo and 3D T2-weighted variable flip-angle turbo spin echo sequences. Eur Radiol 19: 713–721. 18813933 10.1007/s00330-008-1175-7

[5] Kwon JW, Yoon YC, Choi SH (2012) Three-dimensional isotropic T2-weighted cervical MRI at 3T: comparison with two-dimensional T2-weighted sequences. Clin Radiol 67: 106–113. 22142499 10.1016/j.crad.2011.06.011

[6] Naraghi A, White LM (2012) Three-dimensional MRI of the musculoskeletal system. Am J Roentgenol 199: W283–293. 22915419 10.2214/AJR.12.9099

[7] Georgy BA, Hesselink JR (1994) MR imaging of the spine: recent advances in pulse sequences and special techniques. Am J Roentgenol 162: 923–934. 8141019 10.2214/ajr.162.4.8141019

[8] Markl M, Leupold J (2012) Gradient echo imaging. J Magn Reson Imaging 35: 1274–1289. 22588993 10.1002/jmri.23638

[9] Tsuruda JS, Norman D, Dillon W, Newton TH, Mills DG (1990) Three-dimensional gradient-recalled MR imaging as a screening tool for the diagnosis of cervical radiculopathy. Am J Roentgenol 154: 375–383. 2153331 10.2214/ajr.154.2.2153331

[10] Tsuruda JS, Remley K (1991) Effects of magnetic susceptibility artifacts and motion in evaluating the cervical neural foramina on 3DFT gradient-echo MR imaging. Am J Neuroradiol 12: 237–241. 1902019 PMC8331405

[11] van Uijen CM, den Boef JH (1984) Driven-equilibrium radiofrequency ulses in NMR imaging. Magn Reson Med 1(4): 502–7. 6571572 10.1002/mrm.1910010409

[12] Freund PAB, Dalton C, Wheeler-Kingshott CAM, Glensman J, Bradbury D, et al (2010) Method for simultaneous voxel-based morphometry of the brain and cervical spinal cord area measurements using 3D-MDEFT. J Magn Reson Imaging 32: 1242–1247. 21031531 10.1002/jmri.22340 PMC3078516

[13] Melhem ER (2000) Technical challenges in MR imaging of the cervical spine and cord. Magn Reson Imaging Clin N Am 8: 435–452. 10947920

[14] Melhem ER, Itoh R, Folkers PJ (2001) Cervical spine: three-dimensional fast spin-echo MR imaging–improved recovery of longitudinal magnetization with driven equilibrium pulse. Radiology 218: 283–288. 11152816 10.1148/radiology.218.1.r01ja38283

[15] Maldjian C, Adam RJ, Akhtar N, Bonakdarpour A, Boyko OB (1999) Volume fast spin-echo imaging of the cervical spine. Acad Radiol 6: 84–88. 12680429 10.1016/S1076-6332(99)80486-3

[16] Ohshio I, Hatayama A, Kaneda K, Takahara M, Nagashima K (1993) Correlation between histopathologic features and magnetic resonance images of spinal cord lesions. Spine 18: 1140–1149. 8362319 10.1097/00007632-199307000-00005

[17] Takahashi M, Sakamoto Y, Miyawaki M, Bussaka H (1987) Increased MR signal intensity secondary to chronic cervical cord compression. Neuroradiology 29: 550–556. 3124018 10.1007/BF00350439

[18] Kato F, Yukawa Y, Suda K, Yamagata M, Ueta T (2012) Normal morphology, age-related changes and abnormal findings of the cervical spine. Part II: Magnetic resonance imaging of over 1,200 asymptomatic subjects. Eur Spine J 21: 1499–1507. 22302162 10.1007/s00586-012-2176-4 PMC3535246

[19] Vedantam A, Jonathan A, Rajshekhar V (2011) Association of magnetic resonance imaging signal changes and outcome prediction after surgery for cervical spondylotic myelopathy. J Neurosurg Spine 15: 660–666. 21923236 10.3171/2011.8.SPINE11452

[20] Matsuda Y, Miyazaki K, Tada K, Yasuda A, Nakayama T, et al (1991) Increased MR signal intensity due to cervical myelopathy. Analysis of 29 surgical cases. J. Neurosurg 74: 887–892. 1903439 10.3171/jns.1991.74.6.0887

[21] Harrop JS, Naroji S, Maltenfort M, Anderson DG, Albert T, et al (2010) Cervical Myelopathy: A Clinical and Radiographic Evaluation and Correlation to Cervical Spondylotic Myelopathy. Spine 35: 620–4. 20150835 10.1097/BRS.0b013e3181b723af

[22] Morio Y, Teshima R, Nagashima H, Nawata K, Yamasaki D, et al (2001) Correlation between operative outcomes of cervical compression myelopathy and mri of the spinal cord. Spine 26: 1238–1245. 11389390 10.1097/00007632-200106010-00012

[23] Wada E, Ohmura M, Yonenobu K (1995) Intramedullary changes of the spinal cord in cervical spondylotic myelopathy. Spine 20: 2226–2232. 8545717 10.1097/00007632-199510001-00009

[24] Cho YE, Shin JJ, Kim KS, Chin DK, Kuh SU, et al (2011) The relevance of intramedullary high signal intensity and gadolinium (Gd-DTPA) enhancement to the clinical outcome in cervical compressive myelopathy. Eur Spine J 20: 2267–2274. 21779859 10.1007/s00586-011-1878-3 PMC3229731

[25] Arvin B, Kalsi-Ryan S, Karpova A, Mercier D, Furlan JC, et al (2011) Postoperative magnetic resonance imaging can predict neurological recovery after surgery for cervical spondylotic myelopathy: a prospective study with blinded assessments. Neurosurgery 69: 362–368. 21471834 10.1227/NEU.0b013e31821a418c

[26] Avadhani A, Rajasekaran S, Shetty AP (2010) Comparison of prognostic value of different MRI classifications of signal intensity change in cervical spondylotic myelopathy. Spine J 10: 475–485. 20494809 10.1016/j.spinee.2010.03.024

[27] Eck JC, Drew J, Currier BL (2010) Effects of magnetic resonance imaging signal change in myelopathic patients: a meta-analysis. Spine 35: E1306–1309. 20938393 10.1097/BRS.0b013e3181e23e62

[28] Shin JJ, Jin BH, Kim KS, Cho YE, Cho WH (2010) Intramedullary high signal intensity and neurological status as prognostic factors in cervical spondylotic myelopathy. Acta Neurochir 152: 1687–1694. 20512384 10.1007/s00701-010-0692-8

[29] Okada Y, Ikata T, Yamada H, Sakamoto R, Katoh S (1993) Magnetic resonance imaging study on the results of surgery for cervical compression myelopathy. Spine 18: 2024–2029. 8272953 10.1097/00007632-199310001-00016

[30] Mehalic TF, Pezzuti RT, Applebaum BI (1990) Magnetic resonance imaging and cervical spondylotic myelopathy. Neurosurgery 26: 217–226. 2308669 10.1097/00006123-199002000-00006

[31] Kohno K, Kumon Y, Oka Y, Matsui S, Ohue S, et al (1997) Evaluation of prognostic factors following expansive laminoplasty for cervical spinal stenotic myelopathy. Surg Neurol 48: 237–245. 9290710 10.1016/s0090-3019(97)00166-3

[32] Suri A, Chabbra RPS, Mehta VS, Gaikwad S, Pandey RM (2003) Effect of intramedullary signal changes on the surgical outcome of patients with cervical spondylotic myelopathy. Spine J 3: 33–45. 14589243 10.1016/s1529-9430(02)00448-5

[33] Yukawa Y, Kato F, Yoshihara H, Yanase M, Ito K (2007) MR T2 image classification in cervical compression myelopathy: predictor of surgical outcomes. Spine 32: 1675–1678. 17621217 10.1097/BRS.0b013e318074d62e

[34] Chatley A, Kumar R, Jain VK, Behari S, Sahu RN (2009) Effect of spinal cord signal intensity changes on clinical outcome after surgery for cervical spondylotic myelopathy. J Neurosurg Spine 11: 562–567. 19929358 10.3171/2009.6.SPINE091

[35] Ahn JS, Lee JK, Kim BK (2010) Prognostic factors that affect the surgical outcome of the laminoplasty in cervical spondylotic myelopathy. Clin Orthop Surg 2: 98–104. 20514267 10.4055/cios.2010.2.2.98 PMC2867205

[36] Zhang YZ, Shen Y, Wang LF, Ding WY, Xu JX, et al (2010) Magnetic resonance T2 image signal intensity ratio and clinical manifestation predict prognosis after surgical intervention for cervical spondylotic myelopathy. Spine 35: E396–399. 20393392 10.1097/BRS.0b013e3181c6dbc4

[37] Matsumoto M, Toyama Y, Ishikawa M, Chiba K, Suzuki N, et al (2000) Increased signal intensity of the spinal cord on magnetic resonance images in cervical compressive myelopathy. Does it predict the outcome of conservative treatment? Spine 25: 677–682. 10752098 10.1097/00007632-200003150-00005

[38] Chiewvit P, Tritrakarn S, Phawjinda A, Chotivichit A (2011) Predictive value of magnetic resonance imaging in cervical spondylotic myelopathy in prognostic surgical outcome. J Med Assoc Thai 94: 346–354. 21560843

[39] Morio Y, Yamamoto K, Kuranobu K, Murata M, Tuda K (1994) Does increased signal intensity of the spinal cord on MR images due to cervical myelopathy predict prognosis? Arch Orthop Trauma Surg 113: 254–259. 7946816 10.1007/BF00443813

[40] Kang Y, Lee JW, Koh YH, Hur S, Kim SJ, et al (2011) New MRI grading system for the cervical canal stenosis. Am J Roentgenol 197: W134–140. 21700974 10.2214/AJR.10.5560

